# Determining causal miRNAs and their signaling cascade in diseases using an influence diffusion model

**DOI:** 10.1038/s41598-017-08125-4

**Published:** 2017-08-15

**Authors:** Joseph J. Nalluri, Pratip Rana, Debmalya Barh, Vasco Azevedo, Thang N. Dinh, Vladimir Vladimirov, Preetam Ghosh

**Affiliations:** 1 Department of Computer Science, School of Engineering, Virginia Commonwealth University, Richmond, Virginia USA; 2 Center for Genomics and Applied Gene Technology, Institute of Integrative Omics and Applied Biotechnology, Purba Medinipur, West Bengal India; 3 Laboratório de Genética Celular e Molecular, Departamento de Biologia Geral, Instituto de Ciências Biológicas (ICB), Universidade Federal de Minas Gerais, Av. Antonio Carlos,6627, Pampulha, Belo Horizonte, Minas Gerais Brazil; 4 Xcode Life Sciences, 3D Eldorado, 112 Nungambakkam High Road, Nungambakkam, Chennai, Tamil Nadu 600034 India; 5 Department of Psychiatry, Virginia Institute for Psychiatric and Behavioral Genetics, Virginia Commonwealth University, Richmond, Virginia USA; 6 Department of Physiology & Biophysics, Virginia Commonwealth University, Richmond, Virginia USA; 7 Lieber Institute for Brain Development, Johns Hopkins University, Baltimore, Maryland USA; 8 Center for Biomarker Research and Precision Medicine, School of Pharmacy, Virginia Commonwealth University, Richmond, Virginia USA

## Abstract

In recent studies, miRNAs have been found to be extremely influential in many of the essential biological processes. They exhibit a self-regulatory mechanism through which they act as positive/negative regulators of expression of genes and other miRNAs. This has direct implications in the regulation of various pathophysiological conditions, signaling pathways and different types of cancers. Studying miRNA-disease associations has been an extensive area of research; however deciphering miRNA-miRNA network regulatory patterns in several diseases remains a challenge. In this study, we use information diffusion theory to quantify the influence diffusion in a miRNA-miRNA regulation network across multiple disease categories. Our proposed methodology determines the critical disease specific miRNAs which play a causal role in their signaling cascade and hence may regulate disease progression. We extensively validate our framework using existing computational tools from the literature. Furthermore, we implement our framework on a comprehensive miRNA expression data set for alcohol dependence and identify the causal miRNAs for alcohol-dependency in patients which were validated by the phase-shift in their expression scores towards the early stages of the disease. Finally, our computational framework for identifying causal miRNAs implicated in diseases is available as a free online tool for the greater scientific community.

## Introduction

MicroRNAs (miRNAs) are small non-coding RNAs, which are approximately ~20–22 nt in size. They play a crucial role in regulating gene expression by imperfect base-pairing at the 3′-UTRs of messenger RNAs^1^. miRNAs are commonly considered as negative regulators of gene expression^2^, however it has been shown that they also act as positive regulators of gene expression in some cases^3^. miRNAs possess a complex regulatory mechanism of feed-back and feed-forward regulation whereby they regulate their own expression or other genes′ expressions^4^. Multi-level interactions at miRNA regulome-level include miRNA-mRNA, miRNA-environment factors (e.g. virus, stress and radiation), miRNA-transcription factors and also miRNA-miRNA interactions^5^. A miRNA can modulate hundreds of target genes, thereby potentially regulating several cell processes^6^, biological processes and patho-physiological disorders. Hence, the origins of a vast number of diseases have been linked to miRNA (de)regulations^7^. Such miRNA-disease associations have been widely researched^8, 9^ and various models of prediction^10–12^ and identification^13, 14^ of miRNA-disease associations have been developed to study the specific patterns of their interactions. miRNA-disease associations have also been formulated into several network models and various graph theoretical approaches have been implemented^15–17^ to study them from a network topological perspective.

Furthermore, recent cancer biology studies and tumor genome sequencing approaches have investigated into subclonal levels of a particular tumor and clone-based network analysis^18^. These studies have revealed a deeper insight into the complexity of cancers. As opposed to the traditional view of a tumor consisting of a distinct single clone, recent studies have confirmed that a single tumor can contain more than one clone and many distinct subpopulations of genetic profiles (e.g. cells) or clones can mutually exist^19, 20^. Tools such as ABSOLUTE^21^ and ASCAT^22^ are able to computationally quantify and reconstruct the genetic networks tracing the lineage of the mutation. These subclones may consist of distinct functional modules of miRNA, target mRNAs and their interactions. Hence, construction, modeling and comparing networks of every distinct clone within a tumor can provide insights into the working mechanism among subclones. It has been suggested that miRNAs related to same diseases tend to work together in miRNA clusters^23, 24^. In addition, it has been observed that among multi-factorial diseases like cancer, there exist groups of miRNA clusters known as *superfamilies* that are expressed consistently across many cancer phenotypes and may act as *drivers* of tumorigenesis^25^. The presence of such groups not only suggest the need for coordinated targeting and regulation amongst the miRNAs, but also signify that a few critical miRNAs may direct the global expression patterns; and hence it is likely that therapeutic suppression or activation of expression of any one of the few miRNAs in such groups may compensate for the other participants of the group^25^. Despite such evidence, only one previous study has reported an experimental proof of direct miRNA-miRNA interactions^5^ and very few studies have computationally predicted possible miRNA-miRNA interactions^26^.

Although many studies have identified miRNAs associated with diseases, only a few of those have investigated the (signal) cascading influence/effect of miRNA (de)regulations onto other miRNAs or molecular participants. Despite the wide availability of data regarding a miRNA’s direct/indirect effect on various biological processes, identifying or quantifying their influence remains a challenge. To the best of our knowledge, there has not been any model that simulates the time or an event-driven progression of miRNA (de)regulations leading up to a pathophysiological disorder. It is still unknown *how* (de)regulations of a miRNA impact a disease progression and/or their repression. Understanding the progression of such miRNA-driven signaling cascade in the context of diseases is extremely crucial for identifying (i) the critical miRNAs (as potential biomarkers and *directors* of global expression patterns); and (ii) the key stages in the progression of a disease-state under the influence of miRNAs’ expression.

**Figure 1:**
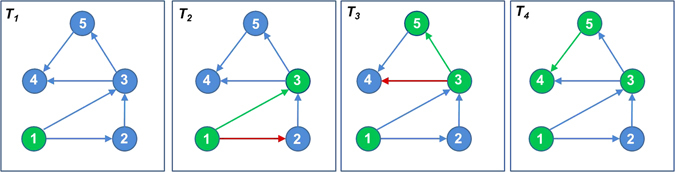
Cascading flow of influence in a *DMIN*.

In this work, we model the passage of miRNA-based influence propagation among other miRNAs as a network diffusion model. We use social behavioral/network principles to model a miRNA’s cascading influence or *flow of information* in and among **d**isease-specific **m**iRNA **i**nteraction **n**etworks *(DMIN)* (elaborated in the *Methodology* section). Essentially, a *DMIN* is a (predicted) miRNA-miRNA interaction network pertaining to a specific disease. These networks often resemble the behavioral characteristics of a social network, such as homophily^27^, wherein participants tend to have positive ties with participants that are similar to themselves; this has already been evidenced in the case of miRNAs^24^. Hence, the application of social network algorithms is apt for modeling the progression of a miRNA’s activity and its signal cascading effect in the context of a disease-state. We explore the property of *information diffusion* through miRNAs which is a crucial characteristic of a *DMIN* network and study the aspect of *information flow* in *DMIN*s. Consider a network of miRNA nodes as shown in Fig. 1. At time point *T*_1_, only *miRNA-1* is activated (in green color). At time point *T*_2_, *miRNA-1* attempts to activate its neighbors, *miRNA-2* and *miRNA-3*. While, *miRNA-2* is not activated (shown by a red arrow), *miRNA-1* successfully activates *miRNA-3* (shown by a green arrow). At time point *T*_3_, *miRNA-3* tries to activate *miRNA-4* and *miRNA-5*, out of which only *miRNA-5* gets activated (shown by a green arrow) while activation of *miRNA-4* is unsuccessful (shown by a red arrow). And at time point *T*_4_, *miRNA-5* successfully activates *miRNA-4*. A particular disease-state is assumed to be highly probable once a required set of crucial miRNA nodes in a network are activated. In this work, we refer to *activating/influencing* a miRNA as a function of time and analogous to affecting a miRNA’s expression and activity. Note that miRNAs implicated in a particular disease may either be up- or down-regulated; our notion of activating/influencing a miRNA is abstract and encompasses both cases. In other words, the nodes in a *DMIN* sequentially activate/influence others where such activation pertains to a significant differential expression of a miRNA over its corresponding expression at control. In this work, we devise a modeling framework to identify the signaling cascade of miRNAs that have already been implicated in particular diseases; our framework can distinguish between causal miRNAs and the affected ones from the global pool of miRNAs that were implicated in a disease. We also present this framework in the form of an online web tool, *miRfluence* that can be readily used by the scientific community. Once the passage of influence between miRNAs is decoded based on the software tool presented here, it will motivate a wide variety of applications ranging from predicting disease progression, disease outcomes and designing drug therapeutics.

## Background

The concept of information diffusion in a network has been widely deployed in the field of social network theory to study spread of ideas, rumors and product adoption between the individuals in the network via the *word of mouth* effect^28–30^. There are essentially two fundamental models of information propagation in social networks - linear threshold *(LT)* and independent cascade *(IC)* model. Every other model proposed in the literature is a derivative of these canonical models. Although, this concept has been applied in the field of sociology to study the various behavioral phenomena, such as the spread of a new concept^31^, it has also been extended to understand the dynamics of spreading of diseases^32–34^. However, understanding influence diffusion in a complex network of miRNAs has never been attempted before and is challenging due to the multi-level nature of interactions. In this work, considering that miRNAs of similar diseases tend to act cooperatively^24^, we focus on the *social nature* of miRNAs related to a class of diseases. We deploy an information diffusion model, through which a miRNA’s influence on its neighboring miRNAs is analyzed and quantified. Social influence can affect a range of behaviors in networks such as dissemination of information/influence, communication and in this case, even mutation. In both the *LT* and *IC* model, the nodes (i.e. the miRNAs) in the network can be in one of the two states - active or inactive. The activated nodes spread their influence by activating their neighboring inactive nodes based on a certain criteria or effect. Garnovetter *et al*.^35^ proposed the *LT* model by applying a specific threshold in each of the nodes of the network. Therein, each node is activated only by its neighbor(s) depending upon the cumulative weight of the incoming edges to the node. The node becomes active when the cumulative sum of the weight of the incoming edges from an active neighboring node crosses its threshold value. Once activated, the node remains active and tries to activate its neighbor, thereby propagating its influence. On the contrary, the *IC* model uses edge probability to determine the information diffusion. In this model, an active node has a single opportunity to activate its neighbors. The edge weights represent the activation probability or likelihood of information propagation in between two nodes. Hence, upon activation, an active neighbor is likely to choose a neighbor with the highest edge weight to activate next.

The miRNA-miRNA interaction network in *DMIN*s used in this study have probability scores as edge weights. These scores act as activation probabilities. Using the *IC* model, upon an activation of a certain miRNA, based on the edge weights between its neighbors, we can determine the next miRNA that is likely to be activated. In this context, activation implies having a causative effect on another miRNA’s expression level. This effect may be direct (when a miRNA directly controls the expression of another one) or indirect (when such regulation can be due to intermediate genes/proteins that these miRNAs regulate). Following this pattern, the information flow or the spread of influence across the miRNAs can be detected. Hence, the pattern of influence across miRNAs in a disease can be identified and studied. Further, we integrate different *DMIN*s belonging to the same category profile, (e.g. ‘gastrointestinal cancers’) and detect the spread of influence among miRNA-miRNA interaction networks belonging to this profile. Subsequently, we determine the key miRNAs playing an influential role among all the diseases within a certain profile.

## Methodology

### Disease-specific miRNA-miRNA interaction networks *(DMIN)*

**Figure 2:**
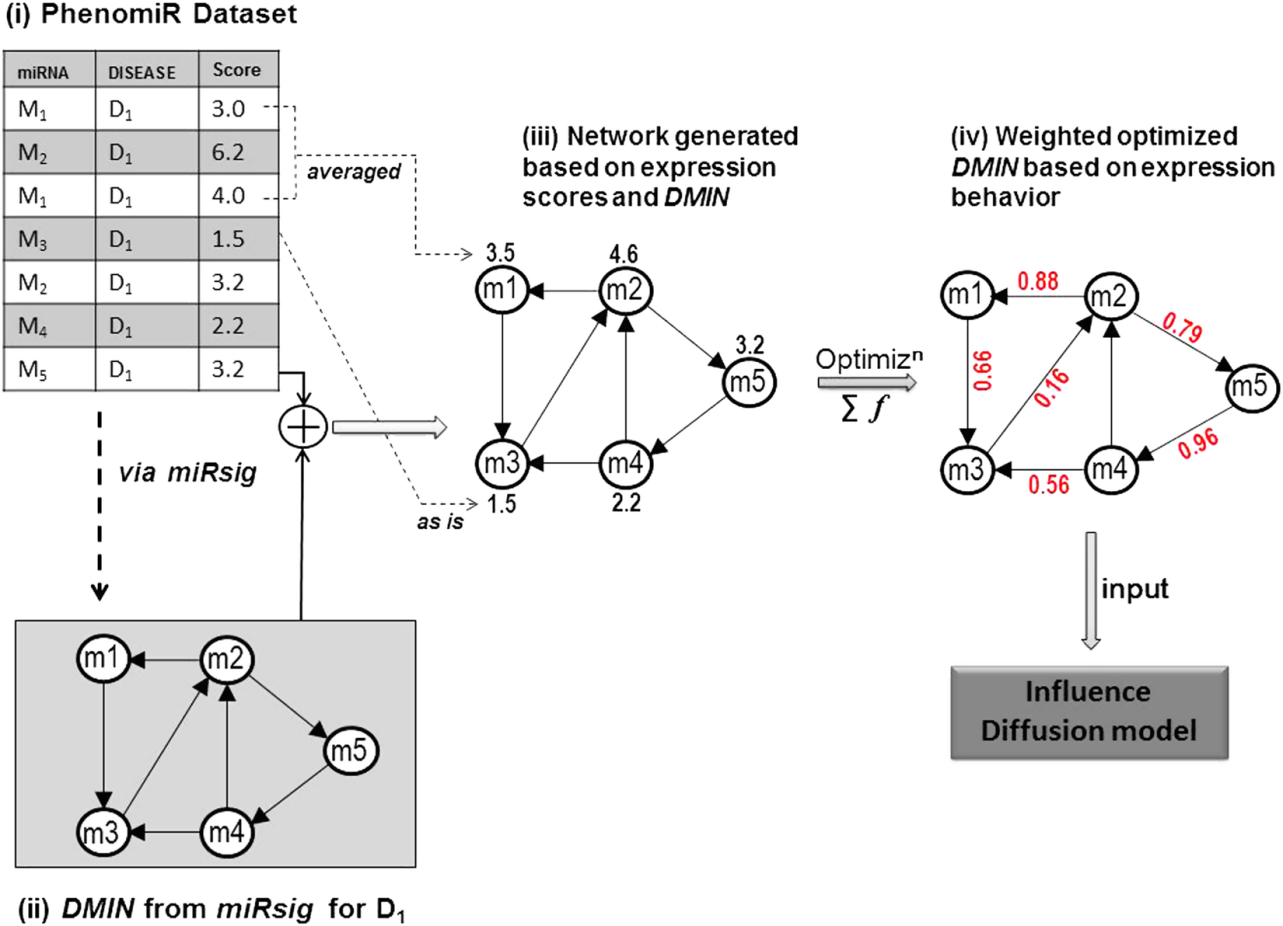
Overview of network generation via optimization of expression scores in a *DMIN*.

*PhenomiR 2*.*0* database^9^ is a manually curated comprehensive data set of differentially regulated miRNA expressions in diseases. It contains 632 database entries collated from 345 articles pertaining to 675 unique miRNAs and 145 diseases. The data curated in PhenomiR is not normalized and is available for download as is. An example of miRNA’s foldchange values and their corresponding regulations in a disease is shown in Fig. 2(i), where miRNAs are denoted by *M*_1_ − *M*_5_ and disease is denoted by *D*_1_. Nalluri *et al*., developed a consensus-based network inference pipeline from the PhenomiR dataset to predict key miRNA signatures (i.e., groups) across several categories of diseases^26^. To briefly summarize this work, they considered a pair of miRNA and disease as a single miRNA-disease (*MD*) entity (or node) which conceptually signifies a disease-specific miRNA. Therefore, the expression score of *M*_*i*_ *D*_*j*_ would mean the expression score of miRNA *i* in disease *j*. Next, they created a miRNA-disease expression matrix, in which the rows represented the various samples/studies and columns represented *MD* nodes. Next, they used six network inference algorithms on the expression matrix and a consensus-based aggregation approach to derive the probabilistic *MD* − *MD* interaction network. From this *MD* − *MD* interaction network, they further extracted **d**isease-specific **m**iRNA-miRNA **i**nteraction **n**etworks (*DMIN*)s and made them available in the tool, *miRsig* ^26^. Further details about this methodology are mentioned in their work^26^. *DMIN*s are directed graphs *G*(*V*, *E*) where *V* is the set of miRNAs being regulated in a specific disease and *E* is the set of weighted edges between them denoting the probability of an interaction. We downloaded the *DMINs* from *miRsig* as is, and further developed an optimization-based methodology (detailed in the next section) to generate a modified *DMIN* which would serve as the input network for the influence diffusion based strategy (Fig. 2(iv)). *miRsig* hosts *DMIN*s for 66 specific diseases. However, to pursue a defined cancer-specific analysis, only 17 *DMIN*s were considered. Based on their tissue-specificity, *DMIN*s were grouped into four categories, namely cancer of the gastrointestinal, endocrine, brain systems and leukemia resulting in four *DMIN*s corresponding to each category.

### Network generation via optimization of expression scores

*DMINs* are directed miRNA-miRNA interaction networks with probability scores as edge weights. To have a network with highest confidence, we extracted *DMIN*s having edge weights of 0.90 score and above. Selecting a high cut-off of 0.9 on the edge scores is a standard practice in such reverse engineering algorithms to ensure confidence in the results. Such algorithms generally suffer from low accuracy due to the noisy expression datasets, non-linearity in the miRNA interactions as well as the high complexity of the inverse problem of inferring *N* ^2^ edges in a network of *N* nodes. Hence, it is customary to work with only the high-confidence edges signified by a 0.9 cut-off on the edge scores. Additionally, the very nature of the influence diffusion set-up works better for sparse graphs; for more dense graphs, most nodes in the network will end up having a high influence score for activating the entire network just because of the availability of more paths to destination nodes. To avoid this possibility, we chose a edge score cut-off of 0.9 in this paper. After deriving these *DMIN*s, we discarded the edge weights (Fig. 2(ii)). We term this network as *DMIN*_*HC*_ (*DMIN* of **h**igh-**c**onfidence). Although, *DMIN*_*HC*_ captures the miRNA-miRNA interaction topology, it does not take into consideration the expression scores of the individual miRNAs within their corresponding diseases (Fig. 2(i)). Expression scores are a vital part of the miRNA-disease regulatory mechanism. Hence, we append *DMIN*_*HC*_ with expression scores for every node (i.e., miRNA), as shown in Fig. 2(iii) resulting in our final network, *DMIN*_*HCE*_(*DMIN* of **h**igh-**c**onfidence and **e**xpression score). The expression scores of miRNAs were converted to their *log*_2_ scores before being incorporated. While incorporating miRNA’s expression scores into *DMIN*, some miRNAs had multiple expression scores for a particular disease (e.g., row #1 and #3 in Fig. 2(i)). In such instances, these multiple scores were *averaged* to get the best possible estimated expression score to be incorporated into the *DMIN*(e.g., node *M*_1_ in Fig. 2(iii)). Nalluri *et al*., demonstrated that *averaging* of the multiple expression scores yielded the best estimate for *DMINs* (*Average Scoring*, under Methods)^26^.

It is important to note that, in our network-building methodology of *DMIN*_*HCE*_, we justify the underlying biological implications of a miRNA’s regulatory behavior. We assume that expression changes in a particular miRNA will have consequential effect on another miRNA’s expression behavior. Hence, if a miRNA has a very high expression score (i.e. degree of fold-change) and is connected to its neighboring miRNAs then it would have a corresponding degree of influence or propagating effect on its neighboring miRNAs. Hence, in order to build a network model which is as close to the underlying biological activity, we design the following optimization-based strategy which provides us with *DMIN*_*HCE*_ with edge-weights, i.e., a weighted *DMIN*_*HCE*_ (see Fig. 2(iv)). These edge-weights would quantify influence of one miRNA onto another, thereby modeling the behavior of miRNA’s regulatory activity based on their expression scores.

#### Optimization formulation for generating edge weights

In order to derive the edge weights for *DMIN*_*HCE*_, the following assumptions were postulated.

1. The direction implies regulatory influence.
2. Each miRNA’s expression score is a cumulative result of its neighboring miRNAs’ expression scores. Hence, the cumulative sum of incoming edge-weights would equal to the expression score of the miRNA. This is denoted by the *Incoming* constraint in the optimization function.
3. Each miRNA’s outgoing edge-weights would not exceed its expression score. A miRNA’s expression score corresponds to its outgoing edge-activity implying that the consequential effect a miRNA has on its neighboring miRNAs is directly correlated to its expression score. However, in this case we introduce a *slack* quantity to make the model more relaxed and feasible for solutions. Without the *slack* variable, the model becomes too restrictive and would not yield any solutions. This is denoted by the *Outgoing* constraint in the optimization function.

The formulation is as follows,

**Objective**: To achieve the most optimal regulatory network flow (i.e., edge weights) characterized by expression scores of each node. This is achieved by obtaining minimum slack (denoted by *s*_*i*_) throughout the network; subject to constraints that (i) the cumulative sum of products of incoming edge-weights and corresponding expression scores of parent nodes would equal the expression score of the target node and (ii) sum of every node’s outgoing edge-weights can exceed its expression score within a *slack* amount.

**Variables**: Let *X*_*i*,*j*_ be the flow of influence from node *i* to node *j*, where *i*, *j* ∈ *n*, and *n* is the total number of nodes in the network, *e* be the fold-change expression of a node, and *s* be the *slack* quantity for ∀ *i*, *j*

**Optimization function:**

1 $$\begin{array}{ll}{\rm{minimize}} & \sum _{i=1}^{n}|{s}_{i}|\\ {\rm{subject}}\,{\rm{to}}\,\mathrm{constraints}: & Incomin{g}_{expression-flow}\sum _{j=1}^{n}{e}_{j}\,\ast \,{X}_{j,i}={e}_{i}(i=1,\,2,\ldots ,n)\\  & Outgoin{g}_{expression-flow}\sum _{j=1}^{n}{e}_{i}\,\ast \,{X}_{i,j}+{s}_{i}={e}_{i}(i=\mathrm{1,}\,\mathrm{2,}\ldots ,n)\end{array}$$

where $$0\le {X}_{i,j};i\ne j$$

The above methodology provided an optimally-weighted *DMIN*_*HCE*_ (Fig. 2(iv)) which was used as the input network for the subsequent influence diffusion algorithm.

The goal of the optimization step is to derive as good an input for the subsequent analysis for influence diffusion based on the expression behavior of miRNAs. Note that ideally for the influence diffusion analysis, the edges should signify the influence of the source node onto the target node. In terms of chemical kinetics of the *A* → *B* edge, such influence is determined by [concentration of the source node A] × [rate constant]; since such rate constants of the miRNA interaction network are unknown (and very difficult to validate experimentally as they comprise indirect interactions of possibly multiple components), we simply considered the influence of an edge to be governed by the concentration of the source node exclusively. Also, considering the steady-state concentrations of the source nodes only signify the equilibrium edge weights and hence the static influence of the source onto the target; this does not capture the time varying influence on the edges as the source node concentration should ideally vary with time.

In addition to the optimization-based network generation method, we also implemented another network generation method - *‘Rescoring all edges to constant weight’*, wherein we assign a constant weight on all the edges of the network. The goal of the more simplistic constant edge weights is to further disregard the steady state source node concentrations and assign equal weightage to all edges in terms of their influence. This formulation can only identify the topological pressure points and should be less accurate. However, due to limitations on the availability of such detailed time-series datasets on miRNA expression levels in specific diseases, it is currently not possible to quantitatively show the difference in accuracy of identifying the influential miRNAs from the two approaches. Perhaps, the common miRNAs that show up to be influential from both approaches will be a better option to consider.

### Influence Diffusion analysis

Upon deriving the weighted *DMIN*_*HCE*_ for 17 diseases and four disease categories, we implemented the influence diffusion algorithms to derive a list of miRNAs ranked according to their highest influence in a disease category (see Section *Compute Influence* and *Algorithm 1*). This algorithm was implemented using the influence maximization code freely distributed^36^.

**Figure 3:**
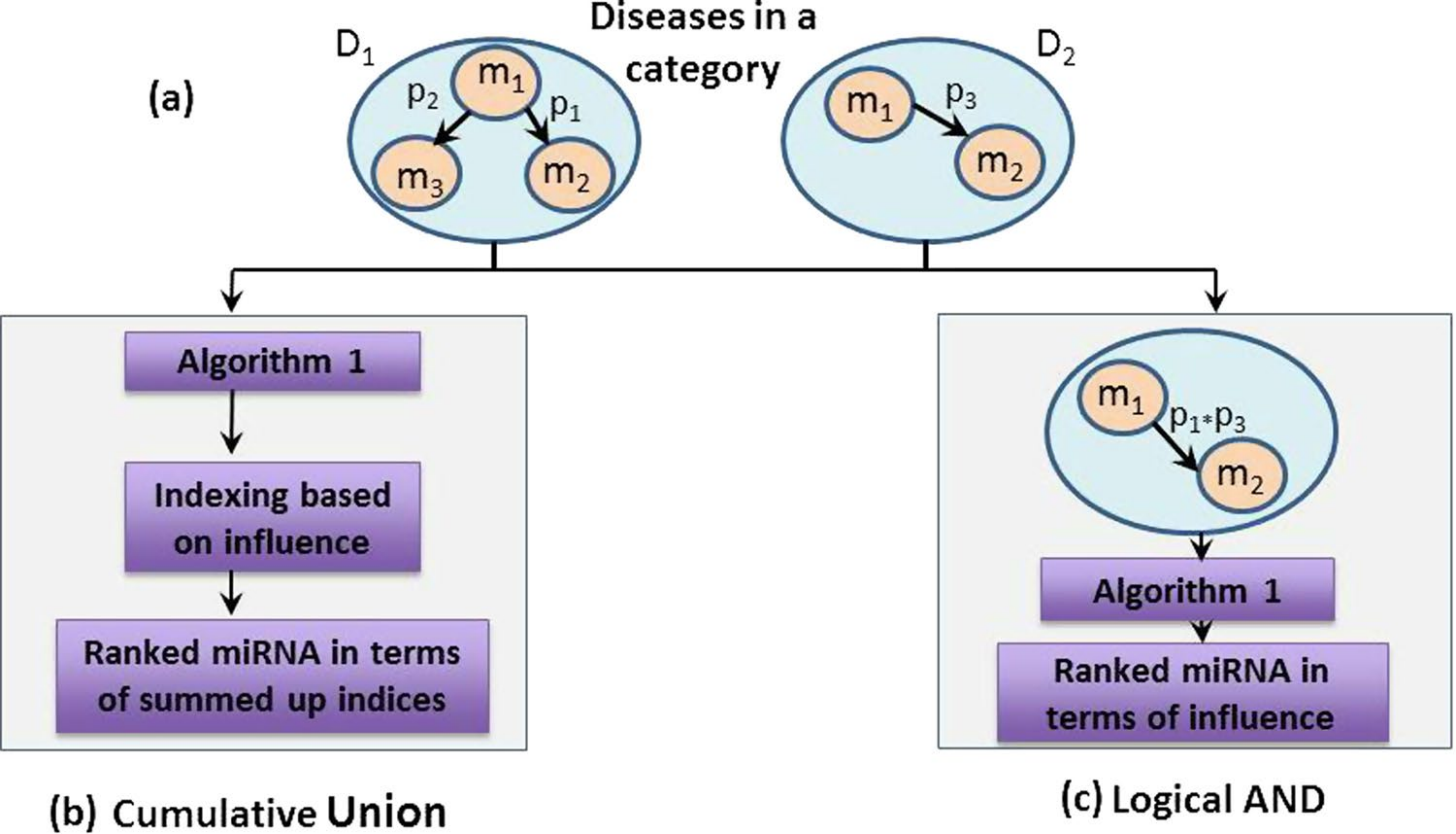
Overview of the workflow of the methodology. Consider two weighted *DMIN*_*HCE*_s belonging to disease *D*_1_ and *D*_2_ which are under the same disease category. The edge *m*_1_ − *m*_2_ is present in both the networks. In the final updated network, the edge weight of *m*_1_ − *m*_2_ is recalculated accordingly using the *Logical AND* operation and upon this updated network, the *Compute Influence* algorithm is implemented.

In a *DMIN*_*HCE*_ of a disease category, there may be multiple occurrences of the same miRNA-miRNA interacting edge due to its presence among several diseases of the category. Here, two approaches are further adopted to calculate their single edge prediction score. As seen in Fig. 3(a), two weighted *DMIN*_*HCE*_s belonging to disease *D*_1_ and *D*_2_ are under the same disease category. The edge *m*_1_ − *m*_2_ is present in both networks with different edge-weights. To address these scenarios, we devised the following two approaches.

(i) Logical AND/Intersection operation
  Under this operation, only the edges which were present in all the diseases of a category were retained in the final disease category network. The edge weights for these common miRNA-miRNA interaction edges were calculated by the following formula,
  2 $${P}_{new}={P}_{1}\times {P}_{2}\times \ldots \times {P}_{n}$$
  where *P*_1_, *P*_2_, and *P*_*n*_ are prediction scores of the same edge in individual disease networks.
  This operation was implemented on the following four categories consisting of the subsequent diseases:
*Gastrointestinal* category: esophageal carcinoma, gastroesophageal carcinoma, gastrointestinal cancer, gastric cancer, colorectal cancer.
*Leukemia* category: hematological tumors, acute myeloid leukemia (AML), susceptibility to chronic lymphatic leukemia, acute myelogenous leukemia.
*Endocrine* category: pancreatic cancer, hepatocellular carcinoma (HCC), thyroid carcinoma (follicular), thyroid carcinoma (papillary).
*Brain systems*: neuroblastoma, medulloblastoma, glioblastoma.
(ii) Cumulative Union:

Under this approach, firstly, for every weighted *DMIN*_*HCE*_ in the category, each miRNA’s coverage was determined using *Algorithm 1*. The coverage value of each miRNA was mapped into a coverage-percentage (e.g., a node having coverage-percentage score of 70 would imply its influence over 70% of the nodes in the network). Next, for the miRNAs which were repeated in multiple diseases within the category - their coverage-percentages were averaged. Finally, the miRNAs are ranked as per their coverage-percentage in the disease category. An explanation of the coverage computation algorithm is provided in the next section.

#### Compute Influence (coverage)

This algorithm (i.e., *Algorithm 1*) is based off of the *IC* model of *information diffusion*. Let *COV*(*u*) denote the coverage/influence of a miRNA node *u* in the network. Upon the execution of the algorithm, all miRNAs are ranked as per their highest coverage/influence. The coverage of each node has been calculated after 10000 monte carlo simulation cycles to achieve the optimal value of coverage.

The algorithmic details of computing the *COV* function are described in the theory of *Independent Cascade* model stated in Kempe *et al*.‘s work^37^; however the following is the summary of its working.

1. Select a node in the network, e.g. consider node 1 in Fig. 4 (*T*_1_).

**Figure 4:**
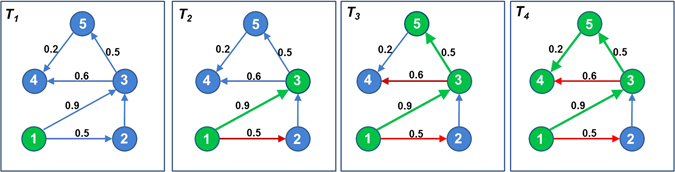
Computation of coverage of influence for node 1. Node 1 activates node 3, node 5 and node 4 based on a series of biased coin-toss operations along its edges.
2. Along its every outgoing edge, perform a biased coin toss, where bias is the edge-probability. In Fig. 4 (*T*_2_), this operation is performed along edges 1 → 2 and 1 → 3 having edge weights 0.5 and 0.9, respectively.
3. If the coin toss operation is successful, then activate the node, and perform step (2) on the newly activated node. In Fig. 4 (*T*_3_), node 2 is not activated (denoted by a red edge, 1 → 2) while node 3 is activated (denoted by a green node 3 and edge 1 → 3). Next, a biased coin toss is performed on node 3 along its edges 3 → 4 and 3 → 5 which results in activation of node 5 and a failed activation of node 4. Subsequently, step (2) is performed on node 5 which results in activation of node 4.
4. Stop when there are no more new activations possible. In Fig. 4 (*T*_4_), no more new activations are possible. Hence, the nodes which can be influenced by node 1 are nodes 3, 5, and 4. Coverage score of node 1 is three.
5. Perform steps (2–4) for the initially selected node (i.e., node 1 in Fig. 4) 10,000 times and finally, average the coverage scores.
6. Repeat steps (1–5) for next node.

**Algorithm 1:**
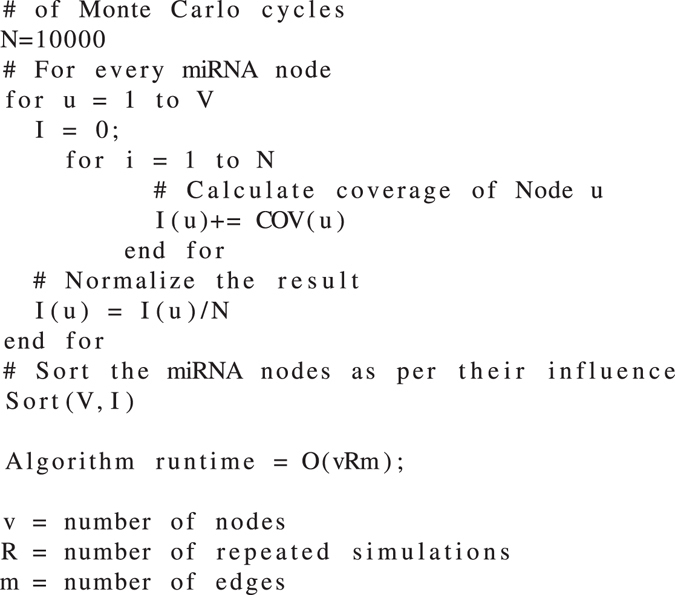
Computing coverage of every node in the network.

## Results

The above methodology was implemented on *DMIN*_*HCE*_s of four disease categories and on individual diseases as well. However, to maintain the emphasis on pan-cancer diseases, we discuss the results of this methodology on the aforementioned disease categories. The results of individual diseases are available and can be downloaded for study and research from the tool *miRfluence*. We implemented the two approaches, i.e., *Intersection/Logical AND* and *Cumulative Union* for the *DMIN*_*HCE*_s of these four disease categories, and the results are labeled under *Influence Maximization* in Table 1. Under the *Cumulative Union* approach, since all the miRNAs (belonging to a category) are ranked as per their coverage percentage, we have selected top 10 miRNAs to be displayed as most influential miRNAs. The results obtained were compared with two other approaches - *miRsig* ^26^ and tool for annotations of miRNAs *(TAM)* ^38^. *miRsig* uses a consensus-based network inference pipeline to predict the crucial miRNAs among the disease categories. The TAM method uses a prediction model to identify novel miRNA interactions and the most likely diseases to be affected (noted with *p*-*values*) for the input set of miRNAs.

**Table 1:** Results of influence maximization methods - *Intersection* and *Cumulative* compared to tools - *miRsig* and *TAM* with relevant PubMed IDs.

Category	Methods					PubMed IDs
	Influence Maximization		miRsig	TAM (disease: p-value)		
	Intersection	Cumulative		Intersection	Cumulative	
Endocrine cancers	hsa-miR-181b-1	hsa-miR-224	hsa-miR-221	thyroid neoplasm: 2.34e-04	thyroid neoplasm: 2.56e-03	18270258
- hepatocellular carcinoma (HCC)	hsa-miR-181a-1	hsa-miR-155	hsa-miR-222	pancreatic: 4.61e-03	pancreatic: 5.76e-04	21139804, 24289824, 16966691
- pancreatic cancer	hsa-miR-224	hsa-miR-222	hsa-miR-155			
- thyroid carcinoma, follicular	hsa-miR-221	hsa-miR-181a-1	hsa-miR-224			
- thyroid carcinoma, papillary	hsa-miR-222	hsa-miR-181b-1	hsa-miR-181a-1			
		hsa-miR-221	hsa-miR-181b-1			
		hsa-miR-187				
		hsa-miR-31				
		hsa-miR-205				
		hsa-miR-181c				
Leukemia cancers	hsa-miR-130a	hsa-miR-126	hsa-miR-29b-1	AML: 1.12e-02	AML: 3.48e03	18337557, 21708028, 19602709
- hematological tumors	hsa-miR-199b	hsa-miR-130a	hsa-miR-20a	CLL: 1.92e-02	CLL: 8.59e-03	17934639, 20439436
- acute myeloid leukemia (AML)	hsa-miR-29b-1	hsa-miR-20a	hsa-miR-126	hematological: 6.53e-03	hematological: 0.127	16192569, 21139804
- chronic lymphatic leukemia (CLL)	hsa-miR-146a	hsa-miR-29b-1	hsa-miR-130a			
- acute myelogenous leukemia	hsa-miR_20a	hsa-miR-99a	hsa-miR-99a			
		hsa-miR-199b	hsa-miR-146a			
		hsa-miR-106a	hsa-miR-199b			
		hsa-miR-146a				
		hsa-miR-222				
		hsa-miR_155				
Gastrointestinal cancers	hsa-miR-181a-1	hsa-miR-29c	hsa-miR-30a	None	Colorectal cancer: 3.06e-02	18607389, 20480519, 22112324
- esophageal	hsa-miR-30a	hsa-miR-181a-1	hsa-miR-181a-1			
- gastroesophageal	hsa-miR-29c	hsa-miR-30a	hsa-miR-29c			
- gastrointestinal		hsa-miR-181b-1				
- gastric		hsa-miR-195				
- colorectal		hsa-miR-221				
		hsa-miR-21				
		hsa-miR-210				
		hsa-miR-99a				
		hsa-miR-126				
Brain systems	hsa-miR-330	hsa-miR-187	hsa-miR-323	None	Glioblastoma: 0.09	17363563, 18577219, 24213470
- neuroblastoma	hsa-miR-149	hsa-miR-181b-1	hsa-miR-129-1		Medulloblastoma: 0.29	18973228, 24213470, 18756266
- medulloblastoma	hsa-miR-331	hsa-miR-137	hsa-miR-137			
- glioblastoma	hsa-miR-107	hsa-let-7a-1	hsa-miR-330			
	hsa-miR-129-1	hsa-miR-150	hsa-miR-149			
		hsa-miR-190	hsa-miR-107			
		hsa-miR-323	hsa-miR-30c-1			
		hsa-miR-107	hsa-miR-181b-1			
		hsa-miR-149	hsa-miR-30b			
		hsa-miR-331	hsa-miR-331			
			hsa-miR-150			
			hsa-let-7a-1			

It is also important to note that there are hardly any tools which predict/determine a list of crucial miRNAs *based on an input set* of diseases. The availability of tools which predict a set of diseases *based on an input set of miRNAs* are also scarce (like tool for annotations of miRNAs(*TAM*)). Many tools provide individual *miRNA-disease* associations and prediction scores but not *set-onto-set* analysis. These factors make *one-on-one* comparison of the proposed methodology very challenging. Hence, we have used the only tools that are available for comparison. The results are presented in Table 1.

1. Endocrine cancers (see row Endocrine cancers in Table 1)
  As per *Logical AND/Intersection* approach, the miRNAs hsa-mir-181b-1, hsa-mir-181a-1, hsa-mir-224, hsa-mir-221 and hsa-mir-222 are key influential miRNAs and they were also predicted as crucial miRNAs as per the tool, *miRsig*. These same miRNAs are also present in the list of top ten miRNAs under the *Cumulative Union* approach. As per the tool *TAM*, all the diseases of this category, i.e., *thyroid neoplasms*, *pancreatic cancer* and *HCC* are very likely to be associated with the aforementioned list of miRNAs. The reported PubMed IDs report the occurrence/expression of all the resultant miRNAs within the same PubMed ID.
2. *Leukemia* (see row *Leukemia cancers* in Table 1)
  Under this category, all the miRNAs determined by the *Logical AND/Intersection* were identified as crucial by the tool *miRsig*. Seven miRNAs predicted by the *Cumulative Union* approach are confirmed by *miRsig*, as well. Among the diseases, acute myeloid leukemia (AML), chronic lymphatic leukemia (CLL) and hematological disorders, were determined as most likely diseases as per *TAM*. The reported PubMed IDs report the occurrence/expression of all the resultant miRNAs within the same PubMed ID.
3. Gastrointestinal cancers (see row Gastrointestinal cancers in Table 1)
  The miRNAs predicted as influential (by *Logical AND/Intersection*) under this category were also predicted to be critical miRNAs by the tool, *miRsig*. The top ten miRNAs predicted by the *Cumulative Union* approach had three of them confirmed by *miRsig* as well. In the gastrointestinal category, *colorectal cancer* is listed in TAM with a *p-value* of 2.03e-3. The reported PubMed IDs report the occurrence/expression of all the resultant miRNAs within the same PubMed ID.
4. Brain systems (see row Brain systems in Table 1)

Under this category, all the miRNAs determined by the *Logical AND/Intersection* appraoch were predicted to be crucial by *miRsig*. Eight out of then reported miRNAs under the *Cumulative Union* approach were corroborated by *miRsig*. *TAM*’s prediction scores for the two diseases (*glioblastoma* and *medulloblastoma*) are not in the confidence margin. However, the reported PubMed IDs report the occurrence/expression of all the resultant miRNAs within the same PubMed ID.

### Case Study and Proof-of-concept

Our proposed methodology is able to identify influential miRNAs in disease-specific networks as demonstrated in the previous section. Furthermore, since the dynamics of miRNA-mediated regulations are similar in biological networks, this methodology has broad applications ranging from networks pertaining to cancers to other pathophysiological conditions, as well. We further demonstrate the application of our proposed methodology on a miRNA expression data set generated from a postmortem brain tissue from patients diagnosed with alcohol dependence (AD).

Tissues for 18 AD patients and matched controls were obtained from a larger sample of 41 AD cases and 41 controls. The postmortem brain sample was received from the Australian Brain Donor Program, New South Wales Tissue Resource Centre, at the University of Sydney, (http://sydney.edu.au/medicine/pathology/trc/). The demographic characteristics of the sample are described elsewhere^39^.

The miRNA expression data were generated using the Affymetrix GeneChip miRNA 3.0 array and normalized using *log*_2_ transformation, followed by quantile normalization, and median-polish probe-set summarization. The final miRNA expression data had 1733 miRNAs and 35 sample tissues (AD- 18, control-17). This expression data is provided in the Supplementary material.

The implementation of our proposed methodology on this data set consisted of the following steps:

1. Construction of a probabilistic miRNA-miRNA interaction network from the miRNA expression matrix based on the *miRsig* pipeline^26^. This network had 1733 miRNAs.
2. From this network, in order to generate a high-fidelity network, we consider only the edges which have a probability score of 0.9 and above.
3. Next, we determine 115 AD-related miRNAs. These miRNAs were derived from a brief literature survey mentioned in Ponomarev’s work^40^ which included miRNAs identified by Sathyan *et al*.^41^, Wang *et al*.^42^, Yadav *et al*.^43^, Lewohl *et al*.^44^ and Nunez *et al*.^45^. We extract a sub-network consisting of 115 miRNAs and the edges among them from the larger network of 1733 miRNAs. This sub-network is a *DMIN* for the disease condition - *alcoholic dependency*.
4. Next, we choose the option of re-scoring the edges of this network with a fixed edge score of 0.01. Note that since this AD dataset involves multiple expression values of each miRNA pertaining to each sample (18 AD and 17 control samples), it is not possible to directly use the optimization formulation for generating edge weights as discussed before; averaging the miRNA expression scores across both control and AD samples will not work here as the AD samples showed significantly different expression levels based on the number of years of alcohol consumption of the patients. Additionally, we did not directly use the edge probabilities from the consensus methodology for generating the miRNA network as such probabilities quantify the *feasibility* of an edge between two miRNAs and not the actual *influence* one miRNA has on the other one. Also, the *DMIN*_*HC*_ is a highly dense and inter-connected network and hence having higher scores of edge weights will cause all the miRNAs to activate its neighbors, thereby labeling all the miRNAs as influential. Moreover, since these edge weights model *regulatory influence and flux phenomena*, lower values are more close to actual biological notion of flux dynamics; note that our goal here is to really understand the topological pressure points in this miRNA interaction network with a constant edge weight of 0.01 on all edges using the influence diffusion model. We tried other (constant) low edge scores as well and the rankings of the miRNAs based on coverage were very similar to the ones obtained here.
5. This *DMIN* is provided as input to the *influence diffusion* model (see *Algorithm 1*).

The result of the above implementation is a ranked list of miRNAs along with their coverage scores. Here, the coverage score implies the number of miRNA nodes that can be activated. For our further comparative analysis we consider the top five miRNAs with highest coverage and bottom five miRNAs with lowest coverage scores. This ranked list of miRNAs is provided in the Supplementary material.

#### Comparative analysis

The influence diffusion phenomena within a miRNA-miRNA interaction network is a time/event-driven progression, characterized by a series of (un)successful activations of miRNA nodes, as explained in Fig. 4. However, the miRNA expression data set of alcohol-dependent patients used in this case study is not a time-series data set. The samples record the *Total years of drinking* alcohol for each patient. The *Total years of drinking* for these 18 samples are - 14, 20, 20, 24, 26, 27, 28, 29, 31, 31, 31, 32, 34, 36, 37, 39, 48 and 48. For the purposes of our modeling and in order to introduce an element of time/event-driven series of progression to the miRNA-miRNA interaction network, we presume these individual samples as time points and observe the expression profiles of the miRNAs in Table 2, across these samples. Our hypothesis is that, these influential miRNAs would have undergone a phase-shift or a distinct change in their expression trend in the beginning stages of the time-points so as to signify an activation moment. This change may correspond to the triggering of the influence diffusion cascade process by the influential miRNAs. On the contrary, the least influential miRNAs would exhibit a similar trend to their control trend with possible phase-shifts occurring only in the later time points.

**Table 2:** miRNAs with the highest and lowest coverage scores after the implementation of *Algorithm 1*.

Category	miRNAs
Top 5 miRs with highest influence	hsa-miR-376c
	hsa-miR-27a
	hsa-miR-30e
	hsa-miR-194
	hsa-miR-9
Bottom 5 miRs with least influence	hsa-miR-196a*
	hsa-miR-606
	hsa-miR-7b*
	hsa-miR-302b*
	hsa-miR-302c*

**Figure 5:**
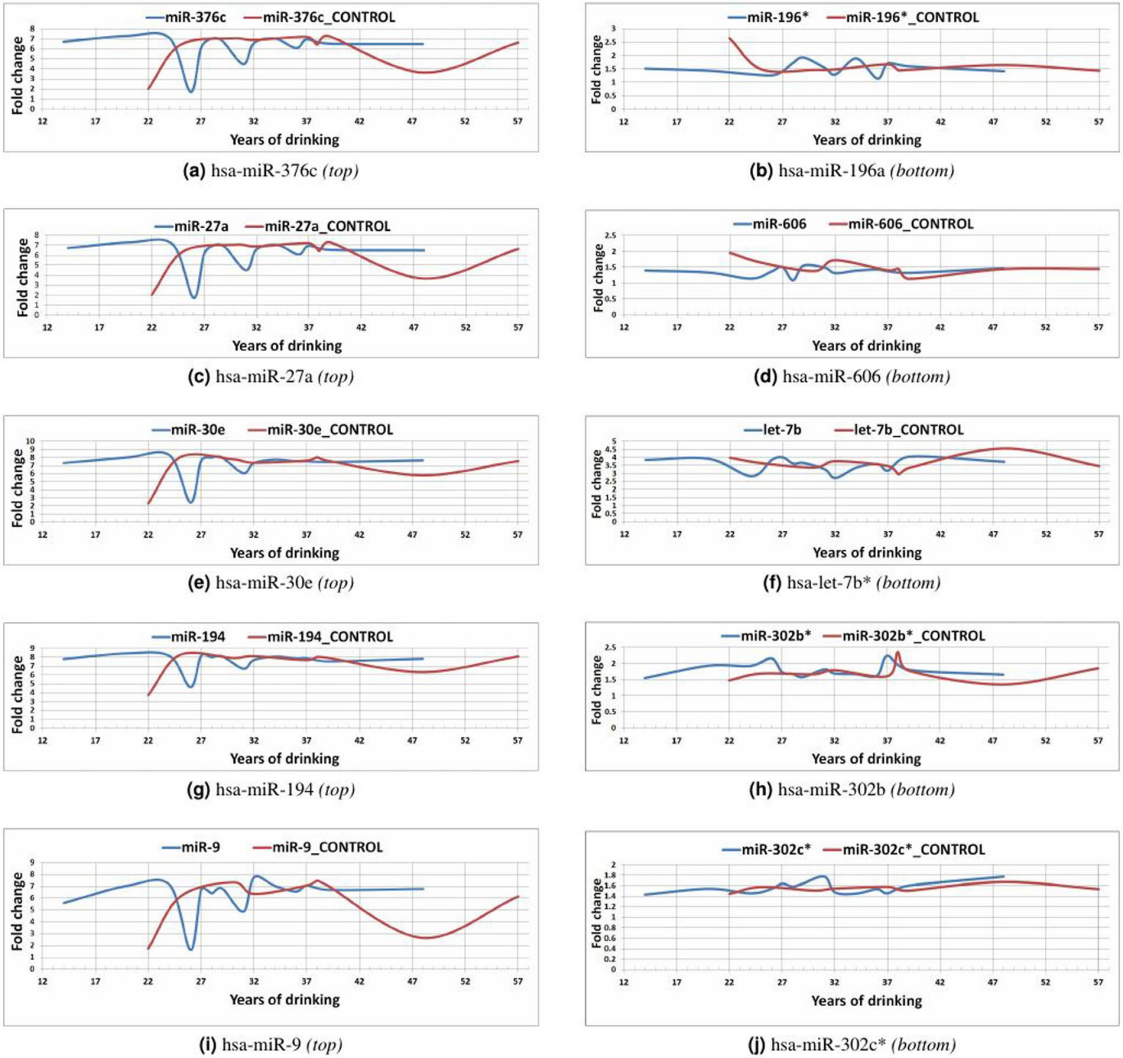
Trendlines of expression scores (AD vs control samples) of miRNAs with highest influence (**a**,**c**,**e**,**g**,**i**) and of miRNAs with lowest influence (**b**,**d**,**f**,**h**,**j**) across sample time points.

We plot the expression scores of these miRNAs (listed in Table 2) against the samples with number of *Total years of drinking*. For same sample time points (such as 20, 31 and 48), we average the expression scores of the miRNAs across AD and *control* samples in order to derive a single time point expression score. The expression trends (*AD* vs *control*) of top five miRNAs are displayed in Fig. 5a,c,e,g,i *(left side)* and those of bottom five miRNAs are displayed in Fig. 5b,d,f,h,j *(right side)*. The expression trends demonstrate that the top 5 miRNAs in AD-samples underwent a phase-shift in the beginning stages (especially around *year* 26) of the time-line when compared to their *control* trend, signifying a triggering of influence diffusion activity within the network. Conversely, the expression trends of the bottom 5 miRNAs in AD-samples align quite well with their *control* trend exhibiting slight fluctuations at later time points. The expression trends and the corresponding data points are provided in the Supplementary material. The results corroborate our earlier stated hypothesis. The expression trends of the top 5 miRNAs also demonstrate that the miRNAs in the AD-samples were operating at a higher expression score from the start, signifying that they were already *activated* and were on an *ON* state. In order to better quantify the differences in their expression trends before and after the phase-shift with respect to the control, we conducted differential expression analysis of these miRNAs using the *limma* package^46^ from R Bioconductor. We performed this analysis across two groups of data set: pre-phase shift and post-phase shift. For the purposes of this analysis, we chose the time-point of *year 26*, as the dividing time-point. The differences in the significance of the expression trends are shown in Table 3. Table 3 demonstrates that the difference in the expression trends of these miRNAs were very significant during the pre-phase shift period with respect to control in comparison to the post-phase shift period. This further emphasizes our hypothesis that the miRNAs underwent a phase-shift signifying the triggering of the influence diffusion cascade process towards the beginning stages of AD.

**Table 3:** Significance of differential expression of top 5 miRNAs *before* and *after* undergoing a phase-shift.

miRNA	Differential expression (p-values)	
	*Pre-phase shift*	*Post-phase shift*
hsa-miR-376c	9.97e-07	0.539
hsa-miR-27a	5.13e-08	0.573
hsa-miR-30e	2.93e-07	0.503
hsa-miR-194	4.62e-06	0.523
hsa-miR-9	1.34e-06	0.829

Pre-phase shift p-values indicate there was a significant difference in the expression of their trends while post-phase shift p-values indicate that the expression trends did not differ significantly, as noted from Fig. 5.

A point to note is that conventional differential expression analysis along with *in-vivo* strategies implicated all 115 miRNAs considered here to play a role in alcohol dependence; so the bottom 5 miRNAs from our list were also implicated in alcohol dependence, albeit we argue that they were more of an *effect* of the signaling cascade, while the top 5 miRNAs exhibit more of a *causal* role.

The expression trends displayed in Fig. 5 of the miRNAs (listed in Table 2) demonstrate that the *influence diffusion* based methodology is able to identify top influential miRNAs playing a causal role in the miRNA interaction network, corroborated quantitatively by the expression trends of these miRNAs across the samples.

### *miRfluence* - an influence diffusion implementation framework

**Figure 6:**
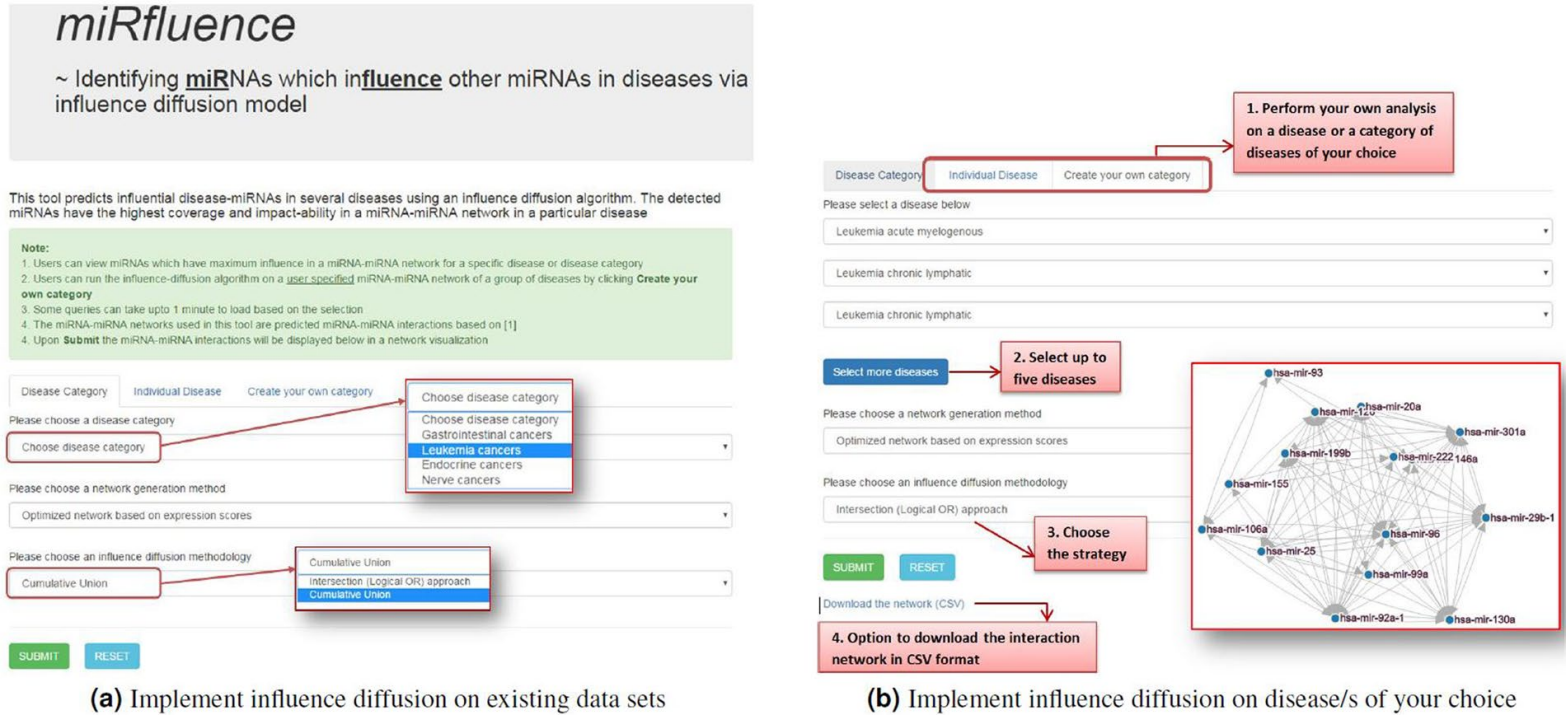
*miRfluence* - an influence diffusion implementation framework.

In order for researchers to implement the proposed influence diffusion methodology on various disease-specific miRNA-miRNA networks or on miRNA networks pertaining to diseases of their interest, we have developed *miRfluence*, an online platform. Using this platform, users can view the influential miRNAs in the miRNA-miRNA networks of existing categories and diseases (Fig. 6a). Users can also implement this methodology on a miRNA interaction network pertaining to any disease of their choice or can also create their own disease category with a combination of up to five diseases (Fig. 6b) from the existing set. Users can view the miRNAs and the topological placement of these miRNAs in the disease network. *miRfluence* also includes two options for identifying the edge weights of the miRNA interaction networks under the *Network generation method* option; these are the (i) optimized network based on expression scores and (ii) rescoring to 0.01 for all the edges considered above the 0.9 cut-off. Users can also choose the two types of influence diffusion implementations described in this work, namely *Logical AND/Intersection* and *Cumulative Union* approach. This tool will help researchers compare/contrast the influence of various miRNAs in similar/contrasting diseases and provide them an insight into the working and grouping of communities of miRNAs in an interactive visualization making comprehension intuitive. The miRNA-miRNA interaction networks can also be downloaded in CSV format which can be easily imported into various network analysis tools for further study and analysis.

*miRfluence* is freely available for research purposes at http://bnet.egr.vcu.edu/mirfluence and has been developed using MySQL as the back-end database and Javascript, PHP, d3.js, AJAX and HTML/CSS for front-end design and visualization.

## Conclusion

In this work, we have implemented the *information diffusion* concept from social networks to identify a crucial set of ranked miRNAs playing an influential role in diseases of a specific profile. Using this methodology, we were able to detect key influential miRNAs in the categories of *Gastrointestinal cancers*, *Leukemia*, *Brain cancers* and *Endocrine cancers*. These results were observed to be significant and were further validated by *miRsig* and *TAM* based analysis. For further validation, we used a miRNA expression data set of patients with alcohol-dependency; our top-ranked miRNAs indeed showed up to have possible causal effects in the miRNA signaling cascade by showing phase-shifts in their expression towards the beginning stages of alcohol consumption in patients.

In our analysis, both the approaches used, i.e., *Logical AND/Intersection* and *Cumulative Union* produced similar results. Among the four categories, with the exception of *Brain cancers* all the miRNAs listed under the *Logical AND/Intersection* approach were included in the top ten ranks of the *Cumulative Union* approach which listed the miRNAs based on highest coverage scores. Hence, a more clear consensus as to which method fared better would emerge by testing these approaches on more comprehensive data sets in the future.

## Electronic supplementary material

Supplementary Dataset 1

Supplementary Dataset 2
